# Clinical Performance and Survival of Bulk-Fill Resin Composites Compared to Conventional Resin Composites in Posterior Permanent Teeth: A Systematic Review and Meta-analysis

**DOI:** 10.7759/cureus.99792

**Published:** 2025-12-21

**Authors:** Abdulaziz Zailai, Ziyad A Alharbi, Fatimah Dowairi, Orjwan Halawi, Shahad A Ghulaysan, Ramis E Safhi, Ola A Mubarki, Raheem F Bashaen, Shatha A Jafari, Wejdan Y Ghazwani, Amal A Muslihi, Manar O Hablool, Amnah A Ageeli, Raghad H Almabdi, Retaj H Jafari

**Affiliations:** 1 Restorative Dentistry, Jazan Specialized Dental Center, Ministry of Health, Jazan, SAU; 2 Dentistry, Ministry of Health, Madinah, SAU; 3 Dentistry, Jazan University, Jazan, SAU; 4 Dental Surgery, Jazan University, Jazan, SAU

**Keywords:** bulk-fill resin composite, clinical performance, incremental layering, meta-analysis, posterior restorations, systematic review

## Abstract

This systematic review and meta-analysis evaluated the clinical performance and survival rates of bulk-fill resin composite restorations compared with conventional incrementally placed resin composites in permanent posterior teeth. A comprehensive search was executed across databases for randomized controlled trials (RCTs) published up to November 2025. The review selected studies comparing direct Class I and II restorations using bulk-fill composites against incremental controls with a minimum follow-up period of 12 months. Restoration failure served as the primary outcome measure. The risk of bias was appraised using the Cochrane RoB 2 tool, and data synthesis employed a random-effects model to compute risk ratios (RR) with 95% confidence intervals (CIs). Nine RCTs involving 632 restorations satisfied the inclusion criteria. Quantitative synthesis indicated no statistically significant disparity in failure rates between the bulk-fill and incremental techniques (RR = 0.82; 95% CI: 0.33-2.01; p = 0.67). Subgroup assessments based on follow-up duration (<24 months versus ≥24 months) yielded no significant variations between the groups. Secondary outcomes, including marginal adaptation, discoloration, and postoperative sensitivity, were comparable across most studies. Bulk-fill resin composites demonstrated clinical durability and survival rates like conventional incrementally placed composites in posterior teeth over short- to long-term periods. The simplified placement protocol of bulk-fill materials provides a time-efficient alternative for posterior restorations without compromising clinical quality.

## Introduction and background

Light-cured resin composites have superseded dental amalgam as the preferred material for restoring posterior teeth, driven by increasing esthetic demands and the preservation of tooth structure inherent to adhesive protocols [1,2]. Despite improvements in material formulations, the longevity of these restorations is threatened by issues such as secondary caries, fractures, and marginal degradation [3,4]. A significant challenge with methacrylate-based composites is polymerization shrinkage. This creates stress at the tooth-restoration interface, compromising marginal integrity and leading to gap formation, microleakage, and eventual failure [5,6]. Also, the mechanical stability of the restored tooth complex is critical, particularly in extensive cavities or endodontically treated teeth, where fracture resistance is compromised [7,8].

The incremental layering technique has been considered the gold standard for placing conventional resin composites to mitigate polymerization shrinkage stress and ensure an adequate depth of cure [9]. However, this technique is clinically demanding and time-consuming as it requires meticulous isolation to prevent contamination between layers and carries the risk of incorporating air voids or inducing bond failure between increments [10,11]. There has been a continuous drive to simplify restorative procedures without compromising clinical outcomes, particularly in complex clinical scenarios, where chairside time is a critical factor for patient management [12,13].

In response to these challenges, bulk-fill resin composites were introduced and designed to be placed in increments of 4-5 mm [14]. These materials incorporate modifications such as increased translucency, novel photoinitiator systems, and polymerization modulators to ensure adequate depth of cure and reduced shrinkage stress [15,16]. Bulk-fill materials are categorized into two distinct types: low-viscosity (flowable) base materials, which require capping with a conventional composite because of their lower wear resistance [11], and high-viscosity (full-body) materials that can be exposed to the oral environment [17]. But recent innovations have expanded this category to include self-adhesive bulk-fill restoratives, which eliminate the bonding step to streamline the procedure [18].

Although recent systematic reviews have synthesized the available data [3,19], the continuous introduction of optimized materials requires an updated analysis of high-quality randomized controlled trials (RCTs) to confirm the long-term performance of these modern composites. Therefore, this systematic review and meta-analysis aimed to evaluate whether bulk-fill resin composites exhibit clinical performance comparable to that of conventional resin composites placed incrementally in permanent posterior teeth, regarding survival rates and restoration quality.

## Review

Methods

Protocol and Registration

This systematic review was conducted according to the Preferred Reporting Items for Systematic Reviews and Meta-Analyses (PRISMA) guidelines [20], and the protocol was registered with the International Prospective Register of Systematic Reviews (PROSPERO) (Registration Number: CRD420251173044).

Eligibility Criteria

The review question was formulated using the PICO framework: "In permanent posterior teeth (P), do bulk-fill resin composite restorations (I) exhibit comparable clinical performance and survival rates to conventional incrementally placed resin composite restorations (C)?"

This review incorporated studies involving patients with vital, permanent posterior teeth (premolars or molars) requiring direct Class I or Class II restorations. The comparison involved bulk-fill resin composites applied in layers of ≥4 mm versus conventional composites placed via an incremental layering technique of ≤2 mm. Success was defined by the absence of restoration failure, specifically the necessity for repair or replacement arising from the fracture, secondary caries, or loss of retention. Also, secondary clinical performance metrics, such as marginal adaptation, color stability, and postoperative sensitivity, were evaluated using standardized systems (USPHS or FDI). The selection criteria were limited to RCTs reporting a follow-up period of at least 12 months.

Studies were excluded if they involved primary teeth, nonvital teeth, or indirect restorations. Also, studies evaluating bulk-fill composites as a liner/base without a control group using conventional incremental composites were excluded, as were in vitro studies, case reports, reviews, and retrospective studies.

Information Sources and Search Strategy

Electronic databases, including PubMed/MEDLINE, Embase, CENTRAL, and Web of Science, were queried using a specific search strategy. Search terms included Medical Subject Headings (MeSH) and free-text variations relevant to "bulk-fill resin", "composite resin", "dental restoration", "clinical trial", and "randomized controlled trial". No limitations were placed on publication year or language. Reference lists of eligible studies and pertinent systematic reviews were manually screened to identify additional articles. The detailed search strings used for each database are provided in Supplementary material 1.

Study Selection and Data Collection

Two independent reviewers screened the titles and abstracts for relevance, as full-text articles of potentially eligible studies were retrieved and assessed against the inclusion criteria, while disagreements were resolved through discussion. Data extraction was performed independently using a standardized form to collect information on the study design, sample size, patient demographics, tooth type, cavity class, adhesive system, composite materials, restoration technique, follow-up duration, evaluation criteria, and clinical outcomes (failure rates and qualitative scores). The standardized data extraction form is available in Supplementary material 2.

Risk-of-Bias Assessment

The risk of bias for each included RCT was assessed using the Cochrane Risk of Bias 2.0 (RoB 2) tool [21], as the domains assessed included the randomization process, deviations from the intended interventions, missing outcome data, outcome measurement, and selection of the reported results. Studies were classified as having "low risk,” "some concerns,” or "high risk" of bias. A detailed rationale for each judgment is provided in Supplementary material 3.

Data Synthesis and Analysis

Data analysis was conducted using R software, Version 4.5.1 (R Foundation for Statistical Computing, Vienna, Austria, https://www.R-project.org/) using the "meta" and "metafor" packages. For dichotomous outcomes, specifically restoration failure, risk ratios (RRs) with 95% confidence intervals (CIs) were computed. Due to anticipated heterogeneity in clinical methodology and materials, a random-effects model (Mantel-Haenszel method) was used. Heterogeneity was assessed using Cochran's Q test and the I² statistic. Subgroup analyses were planned based on the follow-up duration (short-term < 24 months vs. long-term ≥ 24 months). To include studies with zero events in the calculation of the pooled effect size, a continuity correction of 0.5 was applied to all cells of the 2 x 2 table for such studies. Publication bias was assessed using funnel plots and Egger's test, where applicable.

Results

Study Selection

**Figure 1:**
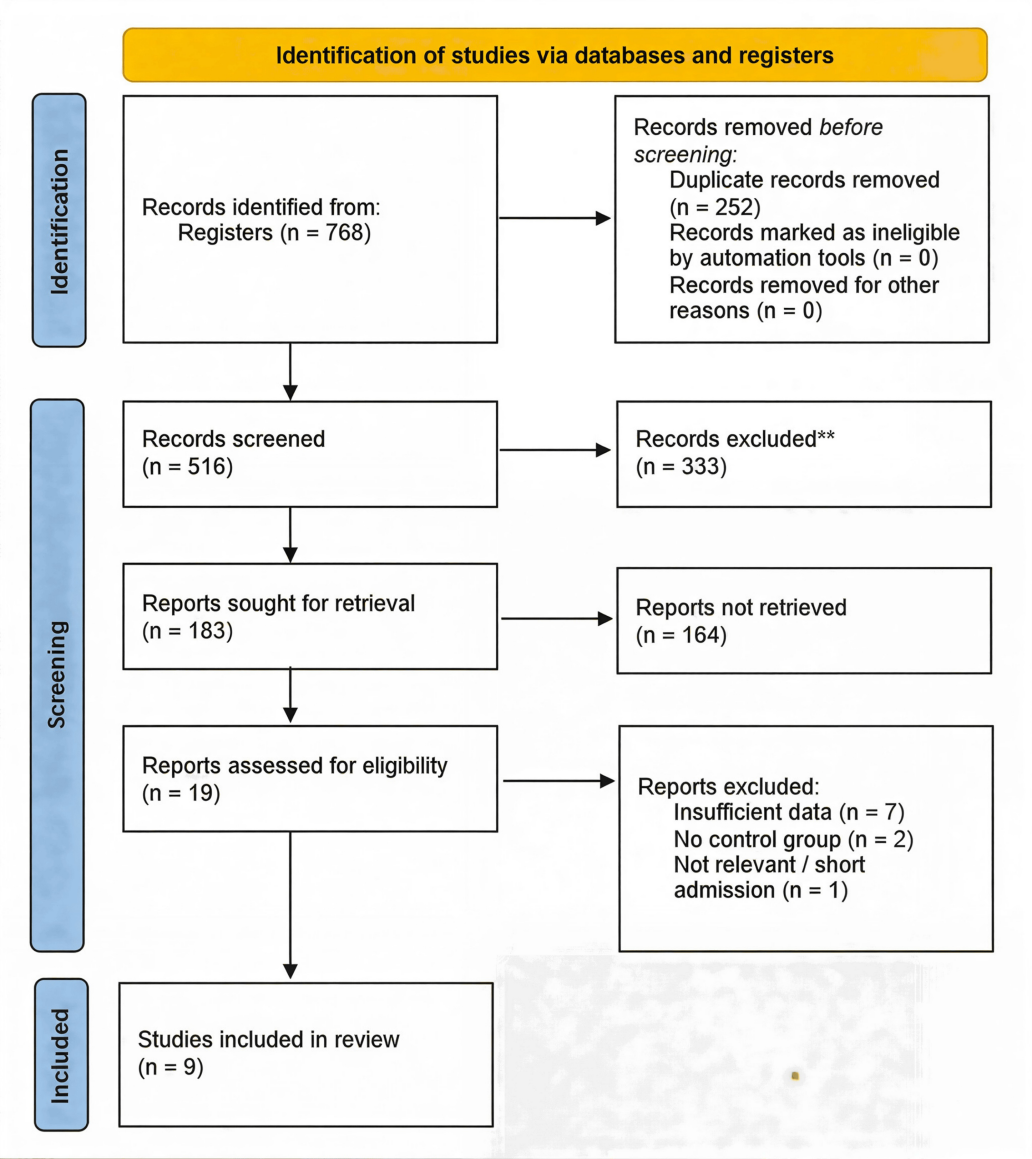
PRISMA flowchart of the study selection process

The initial database query yielded 768 citations. Following the removal of duplicates, 516 records remained. After title and abstract screening, 183 manuscripts were retrieved. Of the 19 full-text articles assessed for eligibility, 10 were excluded. The primary reasons for exclusion were insufficient data for meta-analysis (n = 7), lack of a proper control group (n = 2), and an ineligible study design (n = 1). Nine RCTs satisfied the inclusion criteria and were incorporated into the systematic review and meta-analysis [22-30]. The study selection process is detailed in the PRISMA flowchart (Figure 1).

Study Characteristics

The included studies were published between 2010 and 2024 and were conducted in various countries. All studies were RCTs employing either a split-mouth design [26,28] or a parallel-group design [21], and the follow-up period ranged from 12 to 72 months. A summary of the study characteristics, including the country, design, and follow-up duration, is provided in Table 1.

**Table 1:** Characteristics of the included studies

Study ID	Year	Country	Study design	Follow-up (months)	Sample size (patients)	Total restorations (N)
Kurdi and Abboud [22]	2016	Syria	RCT (parallel)	12	60	60
Arhun et al. [23]	2010	Turkey	RCT (split-mouth)	24	31	82
Atabek et al. [24]	2017	Turkey	RCT (split-mouth)	24	30	60
Bayraktar et al. [25]	2017	Turkey	RCT (split-mouth)	12	50	200
Çolak et al. [26]	2017	Turkey	RCT (split-mouth)	12	34	74
Correia et al. [27]	2023	Brazil	RCT (split-mouth)	30	77	140
Dindaroğlu and Yılmaz [28]	2024	Turkey	RCT (split-mouth)	24	89	178
van Dijken and Pallesen [29]	2017	Sweden/Denmark	RCT (split-mouth)	72	38	106
Yazici et al. [30]	2017	Turkey	RCT (split-mouth)	72	50	104

Patient Demographics and Procedure Details

The meta-analysis included 632 restorations (316 in the bulk-fill group and 316 in the incremental group). Participants were adults requiring Class I or Class II restorations in permanent posterior teeth (premolars and molars). The materials investigated included high-viscosity bulk-fill composites such as Tetric EvoCeram Bulk Fill, Filtek One Bulk Fill, and Quixfil and flowable bulk-fill bases such as SDR capped with conventional composites. The control groups used conventional resin composites placed using an incremental layering technique (increments ≤ 2 mm). Adhesive strategies varied between studies, including etch-and-rinse and self-etch systems. The detailed procedural information is presented in Table 2.

**Table 2:** Patient demographics and procedure details NCCL: non-carious cervical lesion, BF: bulk fill.

Study ID	Mean age (years)	Tooth type	Cavity class	Bulk-fill material (intervention)	Conventional material (control)	Adhesive system
Kurdi and Abboud [22]	20-50	Premolars/molars	Class II	Tetric N-Ceram Bulk Fill	Tetric EvoCeram	Tetric N-Bond (Etch-and-Rinse)
Arhun et al. [23]	26	Premolars/molars	Class I/II	Quixfil	Grandio	Xeno III (Self-Etch)/Futurabond NR
Atabek et al. [24]	7-16	Molars	Class I	SonicFill	Herculite Ultra	OptiBond All-In-One (Self-Etch)
Bayraktar et al. [25]	25.8	Premolars/molars	Class II	Tetric EvoCeram BF/SonicFill	Clearfil Photo Posterior	Various (Self-Etch & Etch-and-Rinse)
Çolak et al. [26]	33.7	Premolars/molars	Class II	Tetric EvoCeram Bulk Fill	Tetric EvoCeram	AdheSE Bond (Self-Etch)
Correia et al. [27]	14.8	Premolars	NCCL	Filtek Bulk Fill Posterior	Filtek Z350 XT	Clearfil SE Bond (Self-Etch)
Dindaroğlu and Yılmaz [28]	9.6	Molars	Class II	Filtek One Bulk Fill	Clearfil Majesty Posterior	G-Premio Bond (Universal)
van Dijken and Pallesen [29]	55.3	Premolars/molars	Class I/II	SDR (Base) + Ceram X mono	Ceram X mono	Xeno V+ (Self-Etch)
Yazici et al. [30]	22	Premolars/molars	Class II	Tetric EvoCeram Bulk Fill	Filtek Ultimate	Excite F (Etch-and-Rinse)

Risk-of-Bias Assessment

**Figure 2:**
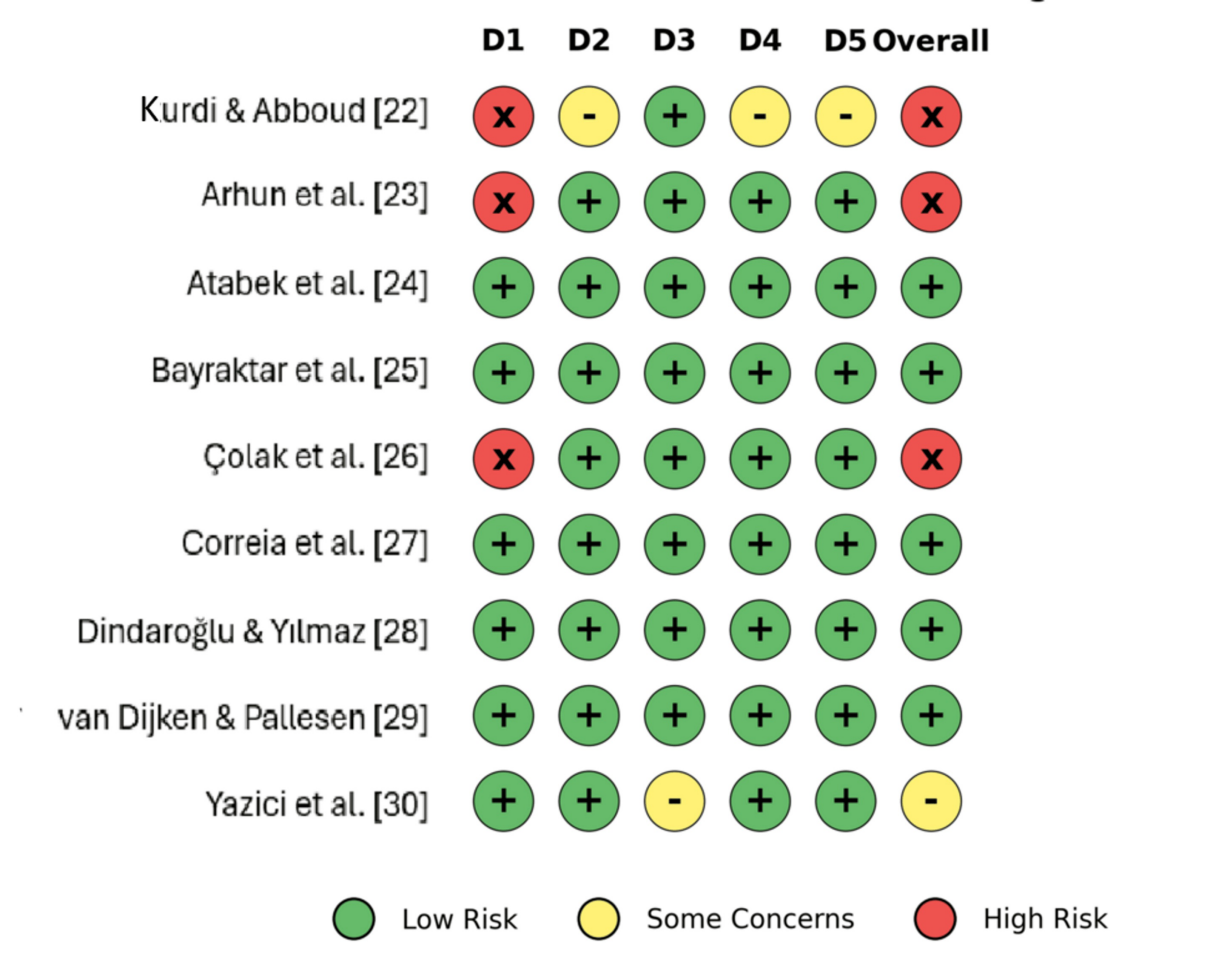
Risk-of-bias assessment (traffic light plot) This figures summarizes the Cochrane RoB 2 tool assessment for each included study across five domains: D1 (bias arising from the randomization process), D2 (bias due to deviations from intended interventions), D3 (bias due to missing outcome data), D4 (bias in measurement of the outcome), and D5 (bias in selection of the reported result). Green (+) indicates a low risk of bias, yellow (-) indicates some concerns, and red (x) indicates a high risk of bias.

**Figure 3:**
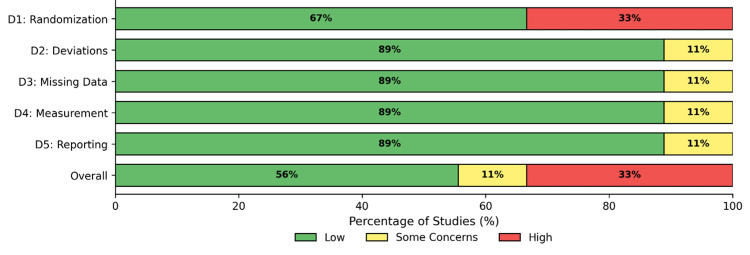
Risk-of-bias summary This figure shows the graphical representation of the proportion of included studies assessed as low risk, some concerns, or high risk of bias for each domain of the Cochrane RoB 2 tool. The domains assessed are "D1: Randomization," "D2: Deviations," "D3: Missing Data," "D4: Measurement," and "D5: Reporting," along with the overall risk of bias.

The risk of bias was assessed using the Cochrane RoB 2. Five studies were classified as "low" risk [24,25,27-29], one study raised "some concerns" due to potential attrition bias [30], and three studies were assessed as "high" risk, due to issues with the randomization process [22,23,26]. The risk-of-bias assessment is shown in Figure 2 and Figure 3.

Meta-analysis of Clinical Performance

**Figure 4:**
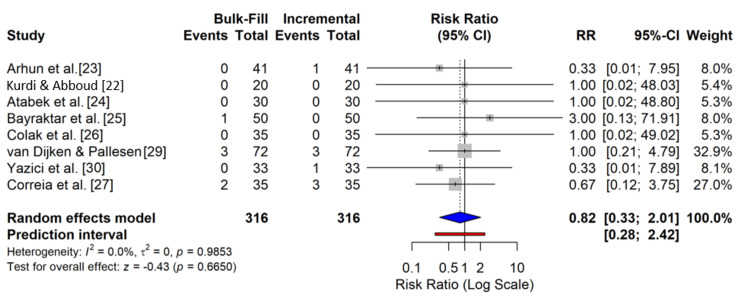
Clinical failure forest plot This plot shows the risk ratio (RR) of restoration failure for bulk-fill versus incremental composites for each study. The squares represent the point estimate of the RR, with the size of the square proportional to the study's weight in the meta-analysis. The horizontal lines represent the 95% confidence intervals (CIs). The diamond represents the pooled RR from the random-effects model. An RR < 1 favors bulk-fill composites, while an RR > 1 favors incremental composites.

The primary outcome, restoration failure, was defined as the need for replacement or repair of the restoration. The overall meta-analysis of the nine included RCTs revealed no statistically significant difference in failure rates between bulk-fill and incremental resin composite restorations (RR = 0.82; 95% CI: 0.33-2.01; p = 0.67). Heterogeneity among the studies was non-existent (I^2^= 0%; p = 0.99), indicating consistent results across different clinical settings and materials. A forest plot illustrating these findings is shown in Figure 4.

Subgroup Analysis

**Figure 5:**
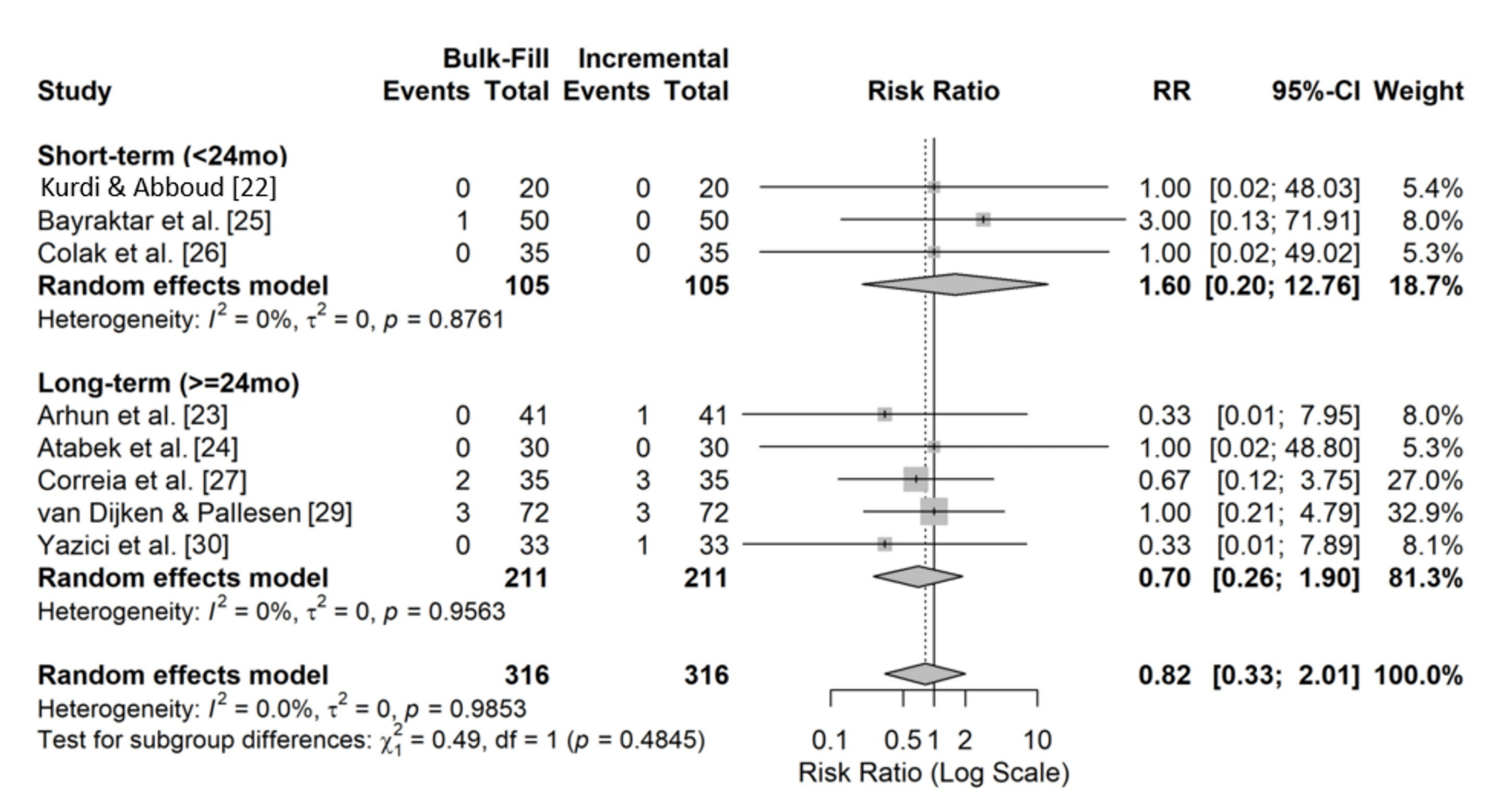
Subgroup forest plot This forest plot shows the meta-analysis of restoration failure rates, stratified by follow-up duration. The studies are divided into two subgroups: "short-term" (<24 months) and "long-term" (≥ 24 months). Each diamond represents the pooled risk ratio (RR) for its respective subgroup. The overall pooled estimate for all studies is shown at the bottom. An RR < 1 favors bulk-fill composites.

A subgroup analysis was performed to account for the variation in study duration based on follow-up time: short-term (<24 months) and long-term (≥24 months), as five studies reported outcomes at 12 months, and the pooled analysis showed no significant difference in failure rates between the two techniques (RR = 1.60; 95% CI: 0.20-12.76; p = 0.66; I^2^= 0%), while four studies reported outcomes at 24-72 months, and no significant difference was observed in the long-term failure rates (RR = 0.70; 95% CI: 0.26-1.90; p = 0.49; I^2^= 0%). The detailed results of the subgroup analysis are presented in Table 3 and Figure 5.

**Table 3:** Subgroup analysis of restoration failure by follow-up duration

Subgroup	Number of studies	Total restorations (bulk-fill)	Total restorations (incremental)	Risk ratio (95% CI)	p-value	Heterogeneity (I^2^)
Short-term (<24 months)	5	180	180	1.60 (0.20, 12.76)	0.66	0%
Long-term (≥24 months)	4	169	168	0.70 (0.26, 1.90)	0.49	0%
Overall	9	349	348	0.82 (0.33, 2.01)	0.67	0%

Publication Bias

**Figure 6:**
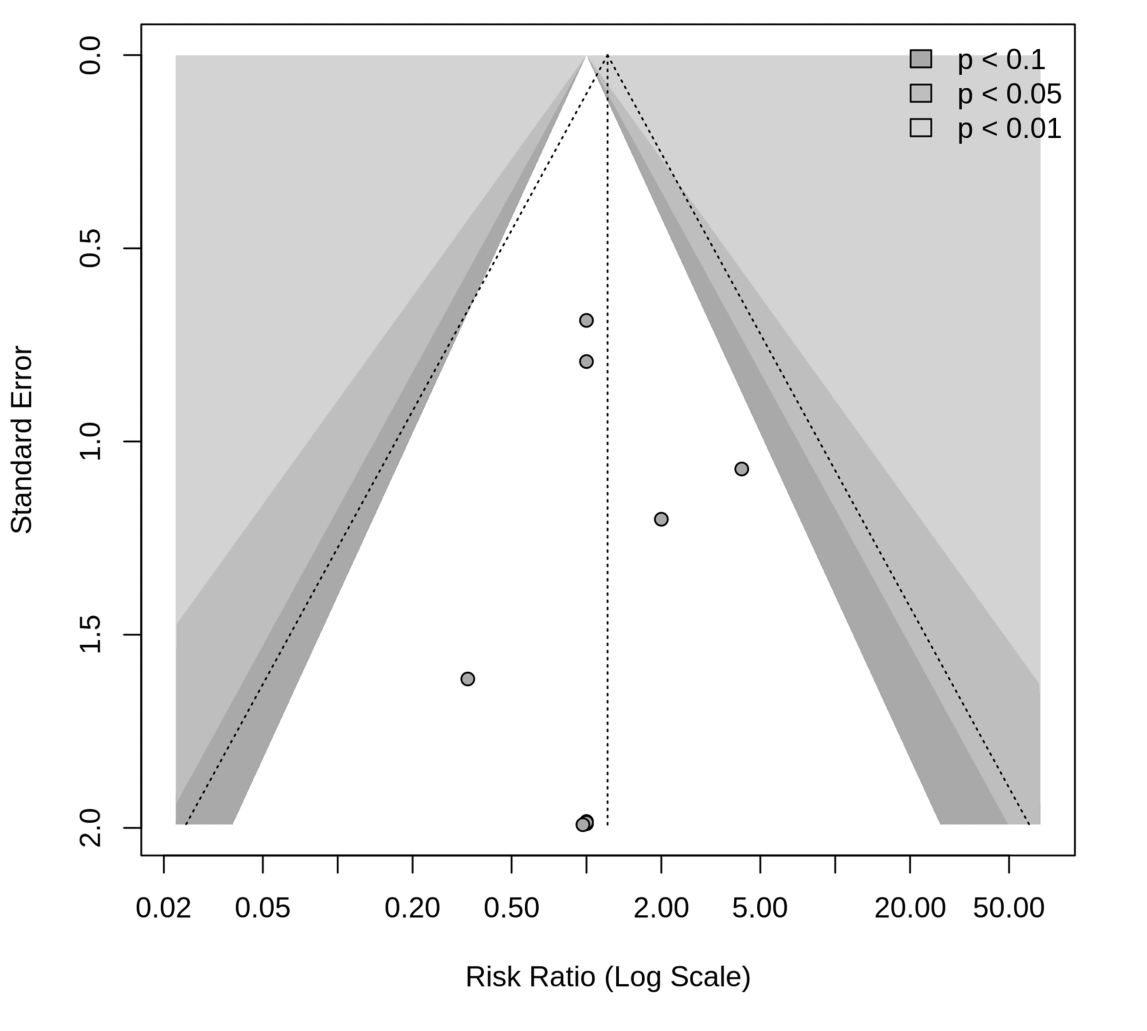
Publication bias funnel plot This plot displays the log risk ratio for each included study against its standard error. In the absence of publication bias, studies are expected to be distributed symmetrically around the pooled effect estimate. Asymmetry can be an indicator of publication bias or other sources of heterogeneity between smaller and larger studies. The contour lines represent the p-values for statistical significance (p < 0.1, p < 0.05, and p < 0.01).

Visual inspection of the funnel plot (Figure 6) revealed a symmetrical distribution of studies, suggesting a low publication bias, but this assessment should be interpreted with caution because of the limited number of studies (<10). Egger's test was not performed because of the small number of studies in the meta-analysis.

Discussion

The current synthesis of data from nine RCTs, encompassing 632 restorations, indicated that there was no statistically significant difference in failure rates between the two restorative protocols (RR, 0.82; 95% CI: 0.33-2.01), which supports the acceptance of the null hypothesis, suggesting that bulk-fill composites possess clinical durability comparable to that of conventional composites when employed in Class I and II cavities.

Clinical Performance and Longevity

Consistent with previous systematic reviews [3,19], the results of this meta-analysis incorporate the latest clinical trial data, thereby strengthening the evidence that supports bulk-fill materials as a viable alternative for posterior restorations. Subgroup analysis confirmed this stability, revealing consistent performance across both short-duration (<24 months) and extended follow-up (≥24 months) periods. The long-term subgroup, which included studies with up to six years of observation [29], demonstrated a low annual failure rate for both material types, underscoring the stability of modern adhesive interfaces and composite resins.

The comparable failure rates may be attributed to the specific stress-relief mechanisms incorporated into bulk-fill composites. Modifications such as polymerization modulators and optimized filler systems allow for a deeper depth of cure and reduced shrinkage stress, even when placed in 4-5 mm increments [14], which counters the theoretical concern that bulk placement might compromise marginal adaptation or increase cuspal deflection compared with incremental layering [8].

Procedural Efficiency and Technique Sensitivity

Procedural efficiency is a primary driver of the adoption of bulk-fill composites, as by eliminating the need for multiple increments, these materials reduce the clinical time and complexity of the restorative procedure [17]. This simplification benefits the clinician and reduces moisture contamination, which is a critical factor in adhesive failure [9]. This review focused on clinical survival, but the comparable performance suggests that the time-saving benefits of bulk-fill materials do not come at the expense of longevity.

Material Variability and Selection

It is important to acknowledge the heterogeneity of the bulk-fill materials included in this review, as the studies evaluated both high-viscosity (full-body) materials such as Tetric EvoCeram Bulk Fill and Filtek One Bulk Fill, and low-viscosity (flowable) base materials capped with conventional composites such as SDR. Despite these differences in viscosity and application mode (single increment vs. base/cap), the overall meta-analysis found no significant deviation in the failure rates, which suggests that both strategies, using a full-body bulk-fill or a stress-breaking flowable base, are effective for posterior restorations. However, clinicians must adhere to the manufacturer’s guidelines regarding capping requirements for flowable variants to ensure adequate wear resistance [11].

Limitations

Although the overall sample size was sufficient for the meta-analysis, the number of studies with long-term follow-up (>5 years) was limited. Furthermore, the subgroup analysis provided a useful exploratory overview of performance across different follow-up durations, but it should be noted that these analyses are statistically underpowered due to the small number of studies within each stratum. The findings from these subgroups should be interpreted with caution until more studies are available to strengthen the evidence. Continued monitoring of ongoing trials is essential to detect potential late-stage failures, such as fatigue fractures or hydrolytic degradation. A meta-regression analysis to explore the influence of study-level covariates (such as specific adhesive strategies or material viscosities) on the treatment effect was considered. However, given the limited number of included studies (n = 9), a meta-regression was inappropriate, as it would be underpowered and could yield unreliable results. Investigating sources of heterogeneity through meta-regression is a key objective for future updates of this review as more high-quality RCTs become available.

Also, the included studies varied in their adhesive strategies (etch-and-rinse vs. self-etch), which introduced a confounding variable, although random-effects modelling was used to account for this. Furthermore, the risk-of-bias assessment highlighted concerns regarding randomization procedures in some older studies, resulting in a high risk of bias classification for three trials. The inclusion of these studies could influence the overall effect estimate, emphasizing the need for rigorous methodological reporting in future research.

Finally, this review included one study by Correia et al. [27] that evaluated restorations in NCCLs. The eligibility criteria focused on Class I and II posterior restorations, but this high-quality RCT was retained as it provides relevant data on the performance of the adhesive-composite interface. However, the different cavity geometry and biomechanical loading in NCCLs introduce a source of clinical heterogeneity. Although the random-effects model was chosen to account for such variability, the results should be interpreted with this inclusion in mind.

## Conclusions

Existing evidence validates the application of bulk-fill resin composites for restoring permanent posterior dentition. They provide clinical performance and survival rates equivalent to those of conventional incrementally placed composites, with the added benefit of a simplified and more time-efficient placement technique.

## Appendices

Supplementary material 1 

**Table 4:** Full electronic search strategies

Electronic databases	Search strategy
PubMed/MEDLINE	(((("Composite Resins"[Mesh]) OR "composite resin*"[tiab] OR "resin composite*"[tiab]) AND ("bulk-fill"[tiab] OR "bulk fill"[tiab])) AND (("Dental Restoration, Permanent"[Mesh]) OR "posterior restoration*"[tiab] OR "Class I"[tiab] OR "Class II"[tiab]) AND ((randomized controlled trial[pt] OR controlled clinical trial[pt] OR randomized[tiab] OR placebo[tiab] OR clinical trials as topic[mesh:noexp] OR randomly[tiab] OR trial[ti])))
Embase	(('composite resin'/exp OR 'composite resin*':ti,ab,kw OR 'resin composite*':ti,ab,kw) AND ('bulk-fill':ti,ab,kw OR 'bulk fill':ti,ab,kw)) AND (('permanent tooth'/exp AND 'dental restoration'/exp) OR 'posterior restoration*':ti,ab,kw OR 'class i':ti,ab,kw OR 'class ii':ti,ab,kw) AND ('randomized controlled trial'/exp OR 'controlled clinical trial'/exp OR 'randomized':ti,ab,kw OR 'placebo':ti,ab,kw OR 'clinical trial':ti,ab,kw)
CENTRAL	([mh "Composite Resins"] OR "composite resin*":ti,ab,kw OR "resin composite*":ti,ab,kw) AND ("bulk-fill":ti,ab,kw OR "bulk fill":ti,ab,kw) AND ([mh "Dental Restoration, Permanent"] OR "posterior restoration*":ti,ab,kw)
Web of Science	TS=("composite resin*" OR "resin composite*") AND TS=("bulk-fill" OR "bulk fill") AND TS=("posterior restoration*" OR "permanent teeth" OR "Class I" OR "Class II") AND TS=(randomized OR randomised OR "clinical trial")

Supplementary material 2 

**Table 5:** Data extraction form

Data field	Description/data to extract
Study information	
Study ID (author, year)	
Country of study	
Study design	(e.g., parallel RCT, split-mouth RCT)
Follow-up duration (months)	
Population characteristics	
Sample size (N patients)	
Total restorations (N)	
Mean age (years)	
Tooth type(s)	(e.g., premolars, molars)
Intervention and comparison	
Cavity class	(e.g., Class I, Class II, both)
Adhesive system (intervention)	(Name, Type: e.g. Etch-and-Rinse, Self-Etch)
Bulk-fill material (intervention)	(product name, manufacturer, viscosity: e.g., high-viscosity, flowable base)
Adhesive system (control)	(Name, type)
Conventional material (control)	(product name, manufacturer)
Outcome data	
Primary outcome: restoration failure	Total events/total N (bulk-fill group)
	Total events/total N (incremental group)
Secondary outcomes (USPHS/FDI)	Scores for: marginal adaptation, discoloration, postoperative sensitivity, etc.
Inclusion/exclusion checklist	Yes/No
Randomized controlled trial?	
Compares bulk-fill vs. incremental?	
Direct restorations in permanent posterior teeth?	
Follow-up ≥ 12 months?	

Supplementary material 3 

**Table 6:** Detailed Risk of Bias 2.0 (RoB 2) judgments for the included studies

Study ID	D1: randomization process	D2: deviations from intended interventions	D3: missing outcome data	D4: measurement of the outcome	D5: selection of the reported result	Overall risk of bias
Kurdi and Abboud (2016)	High risk: method of sequence generation not described; no information on allocation concealment.	Some concerns: no information on blinding of participants or personnel; deviations not reported.	Low risk: outcome data available for nearly all participants.	Some concerns: blinding of outcome assessors not clearly stated.	Some concerns: no protocol available to verify the analysis plan.	High
Arhun et al. (2010)	High risk: randomization by "tossing a coin" does not ensure allocation concealment.	Low risk: participants blinded; no significant deviations from protocol.	Low risk: 100% recall rate at 12 months.	Low risk: examiners were calibrated and blinded.	Low risk: outcomes reported match methods.	High
Atabek et al. (2017)	Low risk: random sequence generation; sealed envelopes used for concealment.	Low risk: appropriate blinding of participants and personnel.	Low risk: low attrition; reasons for missing data balanced.	Low risk: blinded, calibrated examiners.	Low risk: prespecified outcomes reported.	Low
Bayraktar et al. (2017)	Low Risk Random sequence generation; allocation concealment described.	Low Risk Blinding effective; no major deviations.	Low Risk 86% recall rate; missing data unlikely related to outcome.	Low Risk Examiners blinded and calibrated.	Low Risk Results consistent with methodology.	Low
Çolak et al. (2017)	High risk: randomization by "flipping a coin" is inadequate for concealment.	Low risk: participants and personnel blinded.	Low risk: high recall rate; minimal missing data.	Low risk: blinded examiners.	Low risk: all expected outcomes reported.	High
Correia et al. (2023)	Low risk: computer-generated sequence; opaque sealed envelopes used.	Low risk: double-blind design; protocol followed.	Low risk: high recall rate (94.2%); intention-to-treat analysis.	Low Risk Blinded, calibrated examiners.	Low Risk Registered protocol (ReBEC).	Low
Dindaroğlu & Yılmaz (2024)	Low risk: software randomization; allocation concealment used.	Low risk: double-blind design maintained.	Low risk: attrition explained; unlikely to bias results.	Low risk: blinded evaluation.	Low risk: outcomes correspond to registered trial.	Low
van Dijken & Pallesen (2017)	Low risk: predetermined randomization scheme.	Low risk: participants blinded; deviations unlikely.	Low risk: very high recall rate over six years.	Low risk: blinded independent evaluators.	Low risk: standard reporting of all clinical outcomes.	Low
Yazici et al. (2017)	Low risk: random number table; sealed opaque envelopes.	Low risk: double-blind design.	Some concerns: 78% recall at 36 months (>20% attrition); potential for bias.	Low risk: blinded examiners.	Low risk: consistent with trial registration.	Some concerns
